# Elemental Analysis of a Nickel-Titanium (Ni-Ti) Pediatric Rotary File Coated With Graphene Oxide: An Energy Dispersive X-ray Analysis

**DOI:** 10.7759/cureus.73030

**Published:** 2024-11-05

**Authors:** Kuheli Panja, Victor Samuel A, Vivek N, Kavitha Ramar, Rajakumar S, Sujitha Ponraj, Anitha Annadurai, Arya Acca Varghese

**Affiliations:** 1 Pediatric and Preventive Dentistry, Sri Ramaswamy Memorial (SRM) Kattankulathur Dental College and Hospital, SRM Institute of Science and Technology (SRMIST), Chennai, IND; 2 Oral and Maxillofacial Surgery, Sri Ramaswamy Memorial (SRM) Kattankulathur Dental College and Hospital, SRM Institute of Science and Technology (SRMIST), Chennai, IND

**Keywords:** electrophoresis deposition method, energy-dispersive x-ray spectroscopy (edx), graphene nanomaterials, pediatric endodontics, pediatric ni-ti rotary files

## Abstract

Background: Graphene oxide (GO) coatings have emerged as a promising method to enhance materials' surface properties and mechanical performance. In the context of endodontic files, the efficacy of these instruments is critically dependent on the properties of their outermost layer. Surface treatments and coatings can substantially improve these characteristics. GO has been utilized to create nanocomposite coatings to enhance files' surface attributes and mechanical performance.

Aim: This research aimed to study the elemental analysis of a nickel-titanium (Ni-Ti) pediatric rotary file coated with GO using energy dispersive X-ray (EDX) analysis.

Methods: This study used Ni-Ti pediatric rotary files, each 16 mm in length and with an International Organisation for Standardisation (ISO) tip size of #25. Before coating, the existing titanium oxide layer on the files was removed. The GO coating was then applied via electrophoretic deposition (EPD). The chemical composition of the GO-coated endodontic files was analyzed through EDX spectroscopy, and the results were represented in graphical form to evaluate the effectiveness of the coating.

Results: Elemental analysis revealed a significant increase in the weight percentage of carbon (C) across most of the GO-coated files. In contrast, oxygen (O) was more prevalent at the tip and cutting edge, with its weight percentage decreasing along the shaft. These findings indicate the successful deposition of GO on the external surfaces of the coated endodontic files.

Conclusion: GO coatings were effectively applied to Ni-Ti endodontic instruments using EPD. EDX analysis verified uniform deposition of the GO coating across the surface of the Ni-Ti rotary instruments.

## Introduction

Graphene, characterized as a single atomic planar film with a hexagonal honeycomb lattice structure, consists of carbon atoms bonded in an sp2 hybrid orbit. Each carbon atom in this configuration forms covalent bonds with three other atoms through 0.142 nm carbon bonds [1]. Over recent years, graphene has emerged as a highly promising nanomaterial, with its reduced forms attracting considerable interest across various domains, including medicine, electronics, electrical applications, chemical sensors, biosensors, mechanical engineering, and wastewater treatment [2-5]. Surface modification techniques have proven effective in preserving the intrinsic mechanical properties of base materials while imparting new surface characteristics [6,7]. Recently, nanomaterials have garnered significant attention for their potential in material surface modification [8].

Nickel-titanium (Ni-Ti) alloys have revolutionized endodontics since their introduction, offering substantial benefits over traditional stainless-steel files due to their superior mechanical properties. Barr et al. [9] were pioneers in demonstrating the application of Ni-Ti rotary files in primary molars, advocating for the adaptation of biomechanical preparation principles akin to those used for permanent teeth. Numerous studies have documented the clinical success of various rotary file systems, including Profile, Pro Taper, Mtwo, Flex Master, Light Speed LSX, Hero 642, K3, and Wave One, in primary teeth [10,11]. Recently, the Kedo file system, the first rotary pediatric file system, has achieved notable advancements in pediatric endodontics [10]. Despite the advantages of rotary Ni-Ti systems, a significant limitation remains the unexpected fracture of these instruments [12].

Graphene oxide (GO) presents several advantageous properties, including high mechanical strength, photostability, ease of surface modification, and excitation-wavelength-dependent photoluminescence (PL) [13-15]. The high mechanical strength of GO is particularly critical for enhancing the fracture resistance of rotary files. Energy dispersive X-ray (EDX) analysis is a technique employed to measure nanoparticles via scanning electron microscopy (SEM). In this method, nanoparticles are examined using an EDX spectrophotometer, typically integrated into modern SEMs. The nanoparticles are deposited on a suitable substrate that does not interfere with their characterization [16,17].

The elemental analysis of GO-coated Ni-Ti endodontic files has not been extensively investigated till now. The present study aims to provide a comprehensive elemental analysis of GO coatings on the surface of Ni-Ti alloys using EDX analysis. This research offers a straightforward and effective method for the surface modification of dental alloys, thereby providing a reference for advancing the application of GO nanomaterials in medicine and enhancing the properties of dental metals.

## Materials and methods

Tested Ni-Ti rotary instruments

The pediatric rotary Kedo-SG (Reeganz Dental Care Pvt. Ltd., India) rotary files, utilized in this study, are manufactured from heat-treated Ni-Ti using M-wire technology. For this research, Ni-Ti pediatric rotary instruments with a length of 16 mm and an identical International Organisation for Standardisation (ISO) tip size #25 were chosen. The methodology employed for the GO coatings was determined by a study conducted by Panja et al. in 2024 [18]. This selection was made based on the rigorous research and findings of the aforementioned publication.

Procedure for removal of existing coating from rotary instruments

The rotary files tested in this study were initially coated with titanium oxide. To facilitate the deposition of GO, this coating needed to be removed. A solution was prepared by mixing 0.5 ml of medical-grade nitric acid and 0.5 ml of sulfuric acid in 10 ml of water, resulting in a final volume of 100 ml. The Ni-Ti files (N=10) were submerged in this solution for 15 seconds to effectively strip the titanium oxide coating. Following this process, the etched files were thoroughly rinsed with distilled water and subsequently dried in a hot air oven at a temperature of 80°C for 15 minutes.

Electrophoresis deposition method for GO coating on treated rotary instruments

The electrophoretic deposition (EPD) technique was utilized to coat the etched files with GO. In this procedure, a 25 ml beaker was filled with 24 ml of ultrasonicated reduced graphene oxide (rGO) solution. Each of the files (N=10) was immersed in this single-phase rGO suspension. A magnetic stirrer, placed at the bottom of the beaker, maintained continuous agitation at approximately 40 RPM while keeping the temperature constant at 40°C throughout the process. For the deposition, a platinum foil was employed as the cathode, while the etched endodontic file acted as the anode, positioned 15 mm apart. The deposition of GO was conducted for 10 minutes at a constant voltage of 10 V, regulated by an electronic potentiometer. Following the deposition, the files were rinsed with distilled water and subsequently dried at 80°C for 15 minutes.

EDX analysis

Observations of the rotary Ni-Ti instruments were performed using a scanning electron microscope (HR-SEM, Thermo Scientific Apreo S) equipped with EDX (Oxford Inca Energy 350, Oxford Instruments, Abingdon, UK). The pre-coated and GO-coated endodontic file specimens were analyzed at different areas of the file: the tip, the cutting edge, and the shaft. The instruments were carefully mounted on metallic stubs at 10 mm from each other in vertical dimension for EDX analysis with the scanning electron microscope. The instruments had their tip, cutting surface, and shaft examined at 250X magnification, and an elemental analysis was performed. The sequential arrangement on the stubs was: post-etch endodontic file in the first row and GO-coated endodontic file in the second row on the metallic stub, respectively. 

Statistical analysis

The EDX weight percentage values for each key element were analyzed over 11 measurements (N=11) for both pre-coated and post-coated files. Descriptive statistics were employed to determine the mean and standard deviation for each group. For non-parametric distributions, the Wilcoxon signed rank test was conducted using IBM SPSS Statistics for Windows, Version 22 (IBM Corp., Armonk, USA), with the GO coating serving as the distinguishing variable following the EPD process. Statistical significance was evaluated at a 95% confidence level (p<0.05).

## Results

The EDX analysis of GO nanoparticles coated onto Ni-Ti files was conducted to ascertain the weight and atomic percentages of the elements present on the external surface of the files [17]. The EDX spectra identified characteristic peaks corresponding to nickel (Ni), titanium (Ti), carbon (C), and oxygen (O) along the entire length of both the pre-coated and GO-coated endodontic files. Additionally, aluminum (Al) was detected along the shaft of the pre-coated files and throughout the entire length of the GO-coated endodontic files.

**Figure 1:**
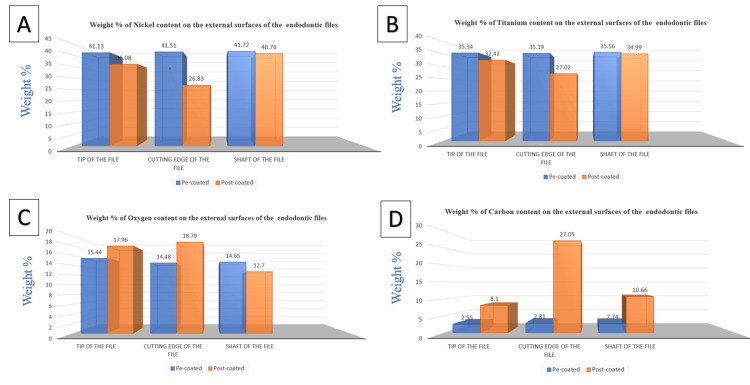
Bar chart showing EDX elemental analysis of different chemical compositions on the external surfaces of the endodontic files denoting (A) nickel, (B) titanium, (C) oxygen, and (D) carbon. EDX: Energy dispersive X-ray analysis

The elemental analysis disclosed that the external surfaces of all examined pre-coated endodontic files were predominantly composed of Ni and Ti, with C and O appearing in lower weight percentages on the tip, cutting edge, and shaft of the files. For the GO-coated endodontic files, while Ni and Ti remained the principal components with a decrease in the weight percentage than the pre-coated file. A notable increase in the weight percentage of C was observed over the greater portion of the file. Contrastingly, O was found in higher weight percentages at the tip and cutting edge of the GO-coated files, but its weight percentage diminished along the shaft (Figure 1).

**Figure 2:**
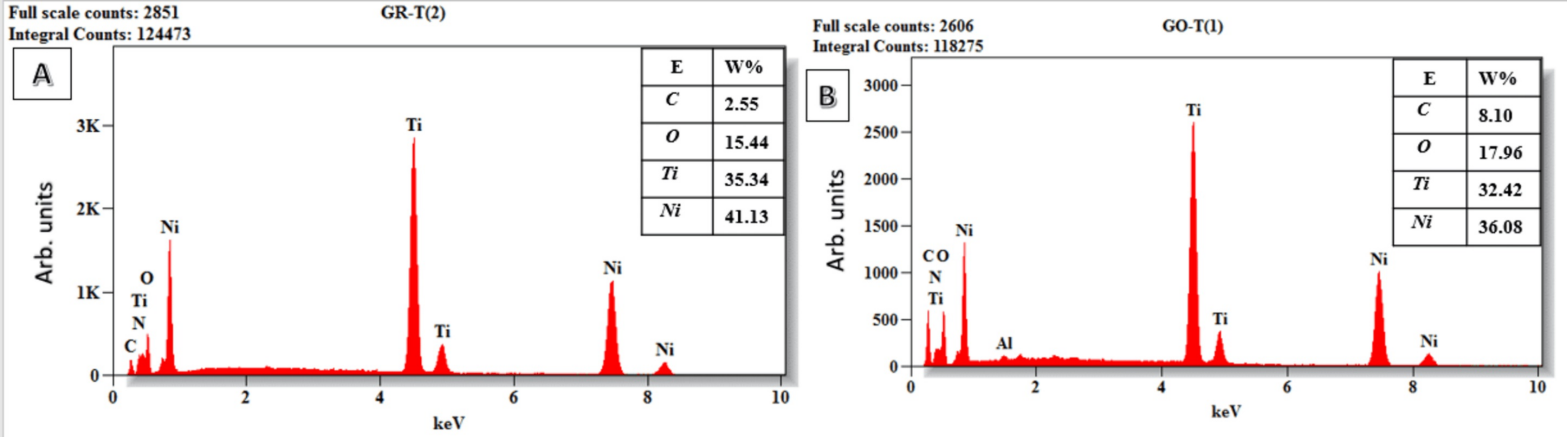
EDX analysis at the tip of the endodontic file. The appropriate elements are displayed. (A) Pre-coated endodontic file. (B) Post-coated endodontic file with GO. EDX: Energy dispersive X-ray analysis; GO: Graphene oxide; Ni: Nickel; Ti: Titanium; O: Oxygen; C: Carbon

**Figure 3:**
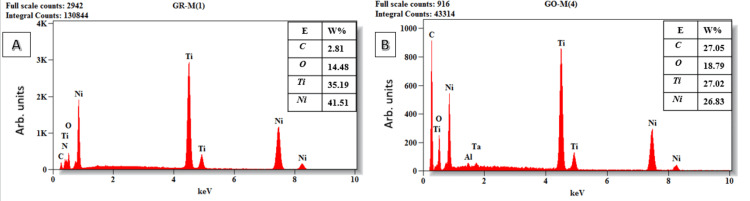
EDX analysis at the cutting edge of the endodontic file. The appropriate elements are displayed. (A) Pre-coated endodontic file. (B) Post-coated endodontic file with GO. EDX: Energy dispersive X-ray analysis; GO: Graphene oxide; Ni: Nickel; Ti: Titanium; O: Oxygen; C: Carbon

**Figure 4:**
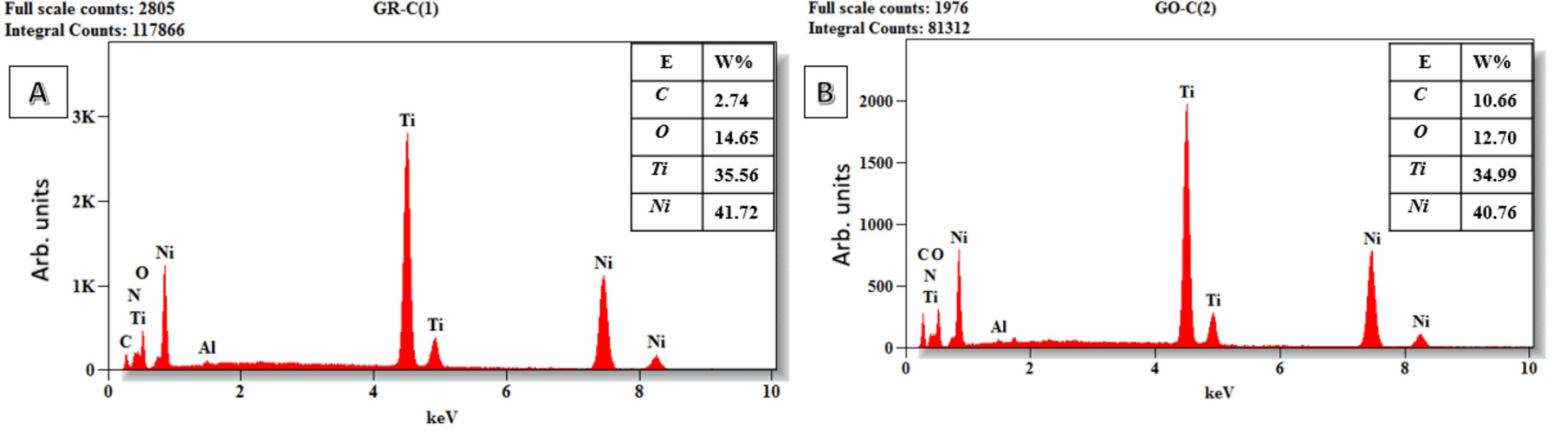
EDX analysis at the shaft of the endodontic file. The appropriate elements are displayed. (A) Pre-coated endodontic file. (B) Post-coated endodontic file with GO. EDX: Energy dispersive X-ray analysis; GO: Graphene oxide; Ni: Nickel; Ti: Titanium; O: Oxygen; C: Carbon

In comparison to the pre-coated endodontic files, the EDX analysis of the external surfaces of the majority of GO-coated endodontic files exhibited higher weight percentages of both C and O. This elevation in the composition of C and O on the coated files signifies the successful deposition of GO on the external surfaces of the GO-coated endodontic files. The EDX analysis also indicates that no detectable amount of Al was introduced into the endodontic file upon the deposition of GO. Consequently, it is highly probable that the Al atoms detected by EDX on the file were not introduced during the deposition process but were inherent impurities of the parent file, as evidenced by their presence on the pre-coated files (Figures 2-4).

The atomic ratio for the GO-coated endodontic files, the carbon/oxygen (C/O) ratio was calculated to be 1.91. This ratio signifies that a substantial level of GO coating was successfully achieved on the surface of the endodontic files. The presence of oxygen-containing functional groups in GO results in a decrease in the C/O ratio.

The Wilcoxon signed rank test indicated a statistically significant alteration in the elemental composition of the files following coating through EPD (Table 1). Specifically, for the GO-coated endodontic files, EDX analysis of the external surfaces demonstrated a notable reduction in the weight percentages of Ni and Ti, accompanied by a marked increase in the weight percentages of C and O relative to the pre-coated files. These changes in elemental composition were statistically significant, confirming the impact of the GO coating on the surface characteristics of the files. A reduction in the oxygen weight percentage was observed only on the shaft of the post-coated files; however, this is of limited significance, as the C content on the shaft was significantly higher than that of the pre-coated files. In summary, the increased weight percentages of C and O, combined with the reduced C/O atomic ratio, conclusively indicate the successful deposition of GO coatings on the endodontic files.

**Table 1:** The EDX values of the elemental composition for pre-coated and post-coated files at the tip, cutting edge, and shaft of the files. *A significant difference was identified among the elements of pre-coated and post-coated files. EDX: Energy dispersive X-ray analysis

Element	Condition	C (mean ± SD)	O (mean ± SD)	Ti (mean ± SD)	Ni (mean ± SD)	p-value (Wilcoxon signed rank test)
Tip	Pre-coated	2.61±0.50	13.76±1.57	35.91±1.87	44.01±2.17	p=0.003*
	Post-coated	8.68±0.43	17.90±0.46	32.29±0.30	36.03±0.466	p=0.003*
Cutting edge	Pre-coated	2.74±0.28	14.45±0.35	35.78±0.60	42.17±0.69	p=0.003*
	Post-coated	27.59±0.50	18.33±0.33	26.35±1.20	26.36±1.20	p=0.003*
Shaft	Pre-coated	2.64±0.17	14.54±0.25	35.99±0.44	41.71±0.41	p=0.003*
	Post-coated	10.81±0.30	12.66±0.30	34.70±0.39	40.50±0.42	p=0.003*

## Discussion

The EDX analysis conducted on the pediatric endodontic files indicated the predominant chemical composition to be Ni, Ti, C, and O. The EPD method employed for applying the GO coating to the endodontic files was executed with high efficacy. This assertion is corroborated by the subsequent EDX analysis performed on the GO-coated endodontic files, which provided confirmatory evidence of the successful deposition process.

The incorporation of Ni-Ti alloys in the production of endodontic instruments has markedly enhanced the efficacy and precision of endodontic treatments [19]. This study concentrates on the specialized pediatric rotary Ni-Ti instruments, which are meticulously crafted from a Ni-Ti alloy, renowned for its flexibility and durability. These instruments are distinguished by a triangular cross-sectional configuration and a blunt tip and help minimize the risk of canal transportation and perforation. Additionally, the files feature a negative rake angle, which is instrumental in efficient cutting action, reducing the likelihood of canal blockage. These files have been selected for their superior performance in achieving high-quality obturation within pediatric root canals [20].

The synthesis of GO films can be accomplished through various sophisticated methodologies, including chemical vapor deposition, EPD, and spin coating. In the context of this investigation, the EPD technique was employed to effectively deposit a GO layer onto endodontic files [21-23]. Through meticulous optimization of laboratory parameters, researchers can modulate the properties of GO to meet specific functional requirements. This capability is of paramount importance for fully exploiting the potential of GO in numerous advanced domains, including electronics, energy storage, catalysis, sensor technology, and biomedical applications [24]. This study examines the interaction between GO and Ni-Ti endodontic files, with a specific focus on the elemental characteristics of the files.

The chemical composition of the GO was meticulously examined through EDX spectroscopy. This analytical technique facilitated the elemental mapping, affirmatively identifying the presence of Ni, Ti, C, and O uniformly distributed across the surface of the endodontic files. The resultant data from the EDX analysis are expressed in weight percent, justified by two principal considerations: Firstly, within the realm of metallurgy, weight percent serves as a prevalent metric for quantifying the gram amounts of each constituent metal in the formation of alloys, as referenced in the source. Secondly, for the sake of consistency and comparability, given that prior research works [25-27] have predominantly reported EDX findings in terms of weight percent, thus aligning our results with established scientific conventions.

Elemental analysis revealed that the endodontic files were predominantly composed of Ni and Ti, findings that align with previous investigations into the surface characteristics of Ni-Ti endodontic files, notably those conducted by Tamer et al. in 2019 [28]. Furthermore, these results are consistent with the elemental composition observed in studies on Ni-Ti substrates by Zinelis et al. in 2010 [25]. For the GO-coated endodontic files, the EDX analysis of the external surfaces demonstrated reduced Ni and Ti weight percentages and elevated weight percentages of both C and O compared to the pre-coated files. These differences were statistically significant and the observations corroborate the findings of Zhang et al. in 2022 [21] and Ma et al. in 2018 [22], where GO was assessed as a surface coating on Ni-Ti substrates. The increased presence of C and O indicates the successful application and integration of the GO coating, thereby validating its efficacy as reported in the aforementioned studies.

The C/O ratio for the GO-coated endodontic files indicated a significant degree of GO deposition on the file surfaces. The ratio obtained in this study is characteristic of GO and differentiates it from pristine graphite, consequently resulting in a lower C/O ratio. The observed value aligns with the findings reported by Chuah et al. in their 2020 study [29], further corroborating the successful incorporation of GO onto the endodontic file surfaces. This quantitative assessment provides valuable insight into the extent of surface modification achieved through the GO coating process, which may have implications for the files' performance and biocompatibility in clinical applications.

However, this study has certain limitations. The thickness of the GO film was not measured using EDX analysis, necessitating further studies involving the files to determine film thickness. Future research could explore the fracture resistance and efficiency of the GO-coated endodontic files.

## Conclusions

The study demonstrates the successful deposition of a homogeneous GO coating on Ni-Ti rotary instruments using EPD. This was validated through comprehensive EDX analysis and the uniform integration of the GO layer was also confirmed. This advancement in dental materials science could enhance the performance and biocompatibility of endodontic rotary instruments in clinical settings.

## References

[1] Li CH, Xiao X, Tao J, Wang DM, Huang CZ, Zhen SJ (2017). A graphene oxide-based strand displacement amplification platform for ricin detection using aptamer as recognition element. Biosens Bioelectron.

[2] Novoselov KS, Fal'ko VI, Colombo L, Gellert PR, Schwab MG, Kim K (2012). A roadmap for graphene. Nature.

[3] Zheng D, Tang G, Zhang HB (2012). In situ thermal reduction of graphene oxide for high electrical conductivity and low percolation threshold in polyamide 6 nanocomposites. Compos Sci Technol.

[4] Peña-Bahamonde J, Nguyen HN, Fanourakis SK, Rodrigues DF (2018). Recent advances in graphene-based biosensor technology with applications in life sciences. J Nanobiotechnology.

[5] Aboelfetoh EF, Elabedien ME, Ebeid EZ (2021). Effective treatment of industrial wastewater applying SBA-15 mesoporous silica modified with graphene oxide and hematite nanoparticles. J Environ Chem Eng.

[6] Ahmed RA, Fadl-allah SA, El-Bagoury N, El-Rab SM (2014). Improvement of corrosion resistance and antibacterial effect of NiTi orthopedic materials by chitosan and gold nanoparticles. Appl Surf Sci.

[7] Bera S, Rout TK, Udayabhanu G, Narayan R (2016). Water-based & eco-friendly epoxy-silane hybrid coating for enhanced corrosion protection & adhesion on galvanized steel. Prog Org Coat.

[8] Zhao J, Xie X, Zhang C (2017). Effect of the graphene oxide additive on the corrosion resistance of the plasma electrolytic oxidation coating of the AZ31 magnesium alloy. Corros Sci.

[9] Barr ES, Kleier DJ, Barr NV (2000). Use of nickel-titanium rotary files for root canal preparation in primary teeth. Pediatr Dent.

[10] Chauhan A, Saini S, Dua P, Mangla R (2019). Rotary endodontics in pediatric dentistry: embracing the new alternative. Int J Clin Pediatr Dent.

[11] George S, Anandaraj S, Issac JS, John SA, Harris A (2016). Rotary endodontics in primary teeth - a review. Saudi Dent J.

[12] Sattapan B, Nervo GJ, Palamara JE, Messer HH (2000). Defects in rotary nickel-titanium files after clinical use. J Endod.

[13] Krishnan SK, Singh E, Singh P, Meyyappan M, Nalwa HS (2019). A review on graphene-based nanocomposites for electrochemical and fluorescent biosensors. RSC Adv.

[14] Daniel A, Oron D, Silberberg Y (2019). Light focusing through scattering media via linear fluorescence variance maximization, and its application for fluorescence imaging. Opt Express.

[15] Boukhoubza I, Khenfouch M, Achehboune M, Mothudi BM, Zorkani I, Jorio A (2019). Graphene oxide/ZnO nanorods/graphene oxide sandwich structure: the origins and mechanisms of photoluminescence. J Alloys Compd.

[16] Yogesh GK, Shuaib EP, Roopmani P, Gumpu MB, Krishnan UM, Sastikumar D (2020). Synthesis, characterization and bioimaging application of laser-ablated graphene-oxide nanoparticles (nGOs). Diam Relat Mater.

[17] Rades S, Hodoroaba V, Salge T (2014). High-resolution imaging with SEM/T-SEM, EDX and SAM as a combined methodical approach for morphological and elemental analyses of single engineered nanoparticles. RSC Adv.

[18] Panja K, A VS, N V, Ramar K (2024). Surface coating of nickel-titanium (Ni-Ti) pediatric rotary file using graphene oxide: a scanning electron microscopy analysis. Cureus.

[19] Elnaghy AM, Elsaka SE (2015). Evaluation of the mechanical behaviour of PathFile and ProGlider pathfinding nickel-titanium rotary instruments. Int Endod J.

[20] Jeevanandan G (2017). Kedo-S paediatric rotary files for root canal preparation in primary teeth - case report. J Clin Diagn Res.

[21] Zhang W, Xu H, Xie F (2022). General synthesis of ultrafine metal oxide/reduced graphene oxide nanocomposites for ultrahigh-flux nanofiltration membrane. Nat Commun.

[22] Ma Y, Han J, Wang M, Chen X, Jia S (2018). Electrophoretic deposition of graphene-based materials: a review of materials and their applications. J Materiomics.

[23] Kamal VV, Arunkumar SS, Kumar KB, Rani SA (2023). Parameter optimization of electrophoretically deposited graphene oxide coating on the frictional characteristics of AISI 52100 alloy steel. Proc Inst Mech Eng Part E J Process Mech Eng.

[24] Esmaeili Y, Bidram E, Zarrabi A, Amini A, Cheng C (2020). Graphene oxide and its derivatives as promising in-vitro bio-imaging platforms. Sci Rep.

[25] Zinelis S, Eliades T, Eliades G (2010). A metallurgical characterization of ten endodontic Ni-Ti instruments: assessing the clinical relevance of shape memory and superelastic properties of Ni-Ti endodontic instruments. Int Endod J.

[26] Testarelli L, Plotino G, Al-Sudani D, Vincenzi V, Giansiracusa A, Grande NM, Gambarini G (2011). Bending properties of a new nickel-titanium alloy with a lower percent by weight of nickel. J Endod.

[27] Al Jabbari YS, Koutsoukis T, Al Hadlaq S, Berzins DW, Zinelis S (2016). Surface and cross-sectional characterization of titanium-nitride coated nickel-titanium endodontic files. J Dent Sci.

[28] Hamdy TM, Galal M, Ismail AG, Abdelraouf RM (2019). Evaluation of flexibility, microstructure and elemental analysis of some contemporary nickel-titanium rotary instruments. Open Access Maced J Med Sci.

[29] Chuah R, Gopinath SC, Anbu P, Salimi MN, Yaakub AR, Lakshmipriya T (2020). Synthesis and characterization of reduced graphene oxide using the aqueous extract of Eclipta prostrata. 3 Biotech.

